# Fs2PA: A Full-Scale Feature Synergistic Perception Architecture for Vehicular Infrared Object Detection via Physical Priors and Semantic Constraints

**DOI:** 10.3390/s26072257

**Published:** 2026-04-06

**Authors:** Boxuan Pei, Leyuan Wu, Xiaoyan Zheng, Chao Zhou, Dingxiang Wang

**Affiliations:** 1 School of Artificial Intelligence, Changsha University of Science & Technology, Changsha 410114, China; pbx6666666@163.com (B.P.); zxiaoyan1998@163.com (X.Z.); zhouchao@hnu.edu.cn (C.Z.); wangdx@hnu.edu.cn (D.W.); 2 State Key Laboratory of Disaster Prevention & Reduction for Power Grid, Changsha University of Science & Technology, Changsha 410114, China

**Keywords:** infrared object detection, full-scale features, gradient injection, autonomous driving, geometric prior

## Abstract

**Highlights:**

**What are the main findings?**

- A Gradient-Informed Attention (GIA) module is proposed to explicitly inject physical geometric priors, successfully breaking the CNN texture bias and overcoming target boundary blurring in thermal crossover scenarios.
- A Full-Scale Feature Synergistic Perception Architecture (Fs2PA) is constructed, utilizing a high-resolution $P_{2}$ layer and a cross-scale shared detection head to simultaneously prevent feature erosion of tiny objects and suppress background noise.

**What are the implications of the main findings?**

- The architecture achieves a state-of-the-art balance between accuracy and efficiency (64.06% mAP@50 and 547 FPS on FLIR v2), proving highly viable for real-time edge deployment in autonomous vehicles.
- It provides a generalizable design paradigm for bridging physical thermal radiation features and deep learning semantic constraints, substantially enhancing all-weather perception robustness.

**Abstract:**

Vehicular infrared object detection is a key technology supporting autonomous driving systems to achieve all-weather environmental perception. However, infrared images inherently lack texture, resulting in blurred object contours. Additionally, deep network propagation severely erodes and loses feature information of distant tiny objects. To address the above issues, this study proposes a Full-Scale Feature Synergistic Perception Architecture for vehicular infrared object detection. This architecture first designs a Gradient-Informed Attention module, which initializes convolution kernels through physical gradient operators to inject geometric prior information into the network, enhancing the model’s perception capability of blurred object boundaries. Secondly, it constructs a Full-Scale Feature Pyramid containing a $P_{2}$ high-resolution feature layer to effectively recover the geometric detail features of distant tiny objects. Finally, it proposes a Scale-Aware Shared Head, which relies on a cross-scale parameter sharing mechanism to achieve extreme parameter compression, and simultaneously introduces deep semantic information to form strong constraints, suppressing noise interference in shallow features. Experimental results on the FLIR v2 and M3FD datasets show that the proposed architecture exhibits excellent detection performance. On FLIR v2, it raises mAP@50 to 64.06% (6.51% relative gain vs. YOLOv11) while maintaining 547 FPS inference speed, achieving an optimal accuracy–efficiency balance.

## 1. Introduction

With the rapid development of advanced driver assistance systems and autonomous driving technology, the robustness of environmental perception systems has become a core element determining vehicle driving safety [1]. Current mainstream environmental perception schemes for autonomous driving are mainly constructed based on visible light cameras and LiDAR (Light Detection and Ranging). However, these types of sensors have significant physical perception blind spots in complex open road scenarios, making it difficult to support all-weather and all-scenario perception requirements. The essence of visible light imaging is relying on external lighting conditions to achieve scene imaging, which makes it extremely prone to imaging failure problems in scenarios such as nighttime or intense lighting changes when entering and exiting tunnels [2,3]. Although LiDAR possesses the technical advantage of active range measurement, in adverse weather environments such as rain, fog, and smoke, the laser beam is easily affected by atmospheric suspended particles, resulting in strong backscattering. This not only causes a sharp decline in detection accuracy but also makes it difficult to effectively extract the semantic features of objects.

In comparison, Thermal Infrared (TIR) sensors operate based on the Blackbody Radiation Law, converting raw thermal radiation in the 8–14 μm band into single-channel intensity maps. Unlike megapixel RGB cameras, uncooled microbolometers in vehicular systems typically suffer from lower native resolutions (e.g., 320×240 or 640×512). Furthermore, constrained by the Noise Equivalent Temperature Difference (NETD), TIR images inherently exhibit elevated thermal noise, array non-uniformity, and a significantly lower Signal-to-Noise Ratio (SNR) [4].

Notwithstanding these noisy and low-resolution characteristics, this passive mechanism endows TIR sensors with two irreplaceable advantages: (1) complete independence from ambient lighting, ensuring robust night-vision target capture in absolute darkness [5]; and (2) superior penetration through atmospheric particles, extending detection ranges in adverse weather like smoke and haze [6]. Consequently, TIR sensors have emerged as a crucial all-weather modality, effectively compensating for the physical perception blind spots of cameras and LiDAR in autonomous driving [3,7].

Despite the significant physical advantages of infrared perception, integrating it with existing deep learning object detection frameworks still poses many technical challenges. Currently, mainstream object detection algorithms (such as the YOLO series [8], Faster R-CNN [9]) are all designed and optimized based on large-scale visible light datasets such as MS-COCO [10] and ImageNet [11]. Related studies show that a convolutional neural network (CNN), after being trained on visible light datasets, exhibits a significant “texture bias”, meaning the model tends to rely on color information and high-frequency texture details to complete object recognition [12]. In contrast, infrared images essentially reflect the temperature distribution characteristics of the scene, naturally lacking color information and being extremely deficient in texture features. This significant difference between the data distribution characteristics and the inductive bias of a CNN results in problems such as feature extraction failure and low object localization accuracy when a standard CNN is directly applied to infrared object detection scenarios [5,12].

The inherent “lack of texture” characteristic of infrared imaging also triggers unique physical detection difficulties, among which the “thermal crossover” phenomenon is a typical thorny problem in vehicular infrared object detection [5]. In actual vehicular driving scenarios, when the surface temperature of the object is close to the ambient background temperature, the radiant temperature difference between the two is extremely small, which causes the edge gradients of the object to almost disappear in infrared images and the visual boundaries of the object become extremely blurred. For such scenarios, pure data-driven CNN models, due to the lack of sufficient texture clues, have difficulty extracting robust discriminative features, making them highly prone to missed detection problems caused by the fusion of the object and the background. This indicates that relying solely on the network’s autonomous learning capability is difficult to solve the problem of blurred features in infrared images, and there is an urgent need to inject physical-level geometric prior information into the network at the early stage of feature extraction, guiding the network to break the “texture bias” and prioritize the faint edge structural features of the objects [13].

If feature blurring is an endogenous defect of infrared images, the contradiction between scale and efficiency brought by the vehicular perspective is a more severe engineering challenge facing vehicular infrared object detection. Affected by the perspective effect of the ego-centric view of vehicular cameras, long-distance objects occupy very few pixels in infrared images, usually less than 16×16 pixels [14]. In existing YOLO series architectures, the feature pyramid is usually constructed starting from the $P_{3}$ layer. The spatial feature information of such long-distance tiny objects will suffer from severe “feature erosion” after multiple convolutional downsamplings, eventually being completely lost in deep feature maps. An intuitive solution is to introduce a higher-resolution $P_{2}$ feature layer to participate in detection to preserve the microscopic detail features of tiny objects, but this strategy has obvious technical drawbacks. On the one hand, the resolution of the $P_{2}$ feature layer is extremely high, and direct introduction will lead to an exponential growth in model computation and memory usage, triggering the “computational overhead” problem and seriously hindering the real-time deployment of the model on resource-constrained vehicular edge chips [15]. On the other hand, shallow $P_{2}$ features lack the constraints of deep semantic information and are easily disturbed by high-frequency background noise in infrared images, leading to problems of “noise amplification” and a surge in false alarm rates. Therefore, how to solve the dual conflict between computational redundancy and background noise interference while effectively preserving the detail features of tiny objects is the core key to achieving efficient vehicular infrared object detection.

To address the above challenges, this study proposes a Full-Scale Feature Synergistic Perception Architecture (Fs2PA) that emphasizes both physical perception and semantic enhancement, achieving a dual improvement in detection accuracy and inference efficiency through the synergistic effect of multiple modules. First, targeting the problem of blurred features in infrared images, a Gradient-Informed Attention (GIA) module is designed in the backbone network, which performs physical initialization of convolution kernels through normalized gradient operators to forcefully inject explicit geometric prior information into the network at the early stage of feature extraction, effectively countering the object feature blurring problem in thermal crossover scenarios. Second, targeting the problem of feature loss for tiny objects from a vehicular perspective, a Full-Scale Feature Pyramid including a $P_{2}$ layer is constructed, while a Scale-Aware Shared Head (SAS-Head) is designed, creating a unique synergistic relationship between the two: the $P_{2}$ layer is responsible for recovering and preserving the geometric features of long-distance tiny objects, while the SAS-Head relies on a cross-scale parameter sharing mechanism to force the leverage of deep semantic information from the $P_{5}$ layer to guide the update and optimization of $P_{2}$ layer features. This design both significantly compresses the number of model parameters, effectively resolving the computational crisis introduced by high-resolution feature layers, and forms strong constraints through deep semantic information, effectively suppressing false alarms caused by shallow background noise.

The main contributions of this study are summarized as follows.

- A physics-guided feature extraction method: To address the problem of blurred features caused by the lack of texture in infrared images, the GIA module is proposed. Through a normalized gradient initialization strategy, explicit geometric priors are injected into the deep network, breaking the “texture bias” of traditional CNN models and significantly enhancing the model’s boundary perception capability in thermal crossover scenarios.
- A Full-Scale feature pyramid construction method oriented towards vehicular scenarios: Addressing the “feature erosion” problem of long-distance tiny objects under the vehicular perspective, a Full-Scale feature architecture including a high-resolution $P_{2}$ layer is constructed, effectively reversing the loss of feature information caused by deep downsampling and ensuring that the features of long-distance tiny thermal source objects are effectively preserved in the feature maps.
- A synergistic lightweight detection head design method: The SAS-Head is proposed to solve the problems of computational redundancy and background noise introduced by the $P_{2}$ layer. This module forms a “Mutual Redemption” relationship with the $P_{2}$ layer: by leveraging a cross-scale parameter sharing mechanism, it not only achieves extreme parameter compression but also forces deep semantics to exert strong constraints on shallow features, effectively suppressing false alarms.
- State-of-the-art vehicular infrared object detection performance: Extensive comparative experiments were conducted on two mainstream infrared object detection datasets, FLIR v2 and M3FD. The results demonstrate that the proposed architecture achieves a significant breakthrough in detection accuracy while maintaining an ultra-high real-time inference speed of 547 FPS, fully validating the effectiveness and superiority of the synergistic design of physical perception and semantic constraints.

The remainder of this paper is organized as follows. Section 2 reviews related works in infrared object detection, multi-scale architectures, and lightweight detection head designs. Section 3 details the overall framework of the proposed Fs2PA, elaborating on the physical mechanisms of the GIA module, the Full-Scale Feature Pyramid, and the SAS-Head. Section 4 presents the experimental setup, comprehensive ablation studies, comparative analyses with state-of-the-art methods, and in-depth discussions on real-world deployment boundaries and future research directions. Finally, Section 5 summarizes the core conclusions and the broader impact of this study.

## 2. Related Works

### 2.1. Infrared Object Detection and Physical Feature Enhancement

In recent years, deep learning has greatly promoted the development of infrared object detection. Most early methods followed the paradigm of transfer learning, directly applying object detectors designed for visible light images (such as Faster R-CNN [9] and the YOLO series [8]) to infrared datasets. Although these methods have achieved certain results through fine-tuning on large-scale infrared datasets, they essentially ignore the physical differences between infrared imaging and visible light imaging. As pointed out by Dai et al. [5], infrared images naturally lack color and fine-grained texture. Directly using standard CNN backbones can easily lead to the failure of feature extractors when facing low-contrast scenarios such as “thermal crossover”.

To compensate for the lack of texture information, some studies have attempted to introduce explicit physical feature enhancement. One mainstream category of methods adopts multi-modal fusion strategies, utilizing visible light images to provide texture details [16], but this introduces complex registration problems and additional hardware costs. Another category of methods attempts to use traditional edge operators (such as Sobel or differential-like operators) to extract gradient maps, which are integrated as additional input channels or feature guidance mechanisms [17]. For example, the Pixel Difference Networks (PiDiNet) proposed by Su et al. [13] integrate traditional gradient operators into convolution operations to enhance edge perception. However, most of these methods belong to “add-on enhancement”: they either require tedious pre-processing steps or need to maintain additional feature extraction branches, significantly increasing the inference latency of the model. Different from the aforementioned methods, the GIA module proposed in this paper adopts an “embedded” strategy: we directly endow the backbone network with physical perception capability through normalized gradient initialization. This design achieves “plug-and-play” of physical priors without introducing extra branches or complex pre-processing, significantly improving the robustness of infrared features without increasing the inference burden.

### 2.2. Small Object Detection and Multi-Scale Architectures

In vehicular infrared perception, the detection of long-distance tiny objects is a core difficult problem. To alleviate the feature loss caused by downsampling in deep networks, Feature Pyramid Network (FPN) [18] and Path Aggregation Network (PANet) [19] are widely used to fuse multi-scale features. In the related field of Infrared Small Target Detection (IRSTD), pioneering architectures like DCCS-Det [20] have proven the efficacy of cross-scale contextual modeling for extracting small thermal targets from backgrounds. Meanwhile, in visible-light aerial scenarios, Zhu et al. proposed TPH-YOLOv5 [21], which achieved significant performance improvements by introducing a Transformer Prediction Head and adding a high-resolution tiny object detection layer. However, the enormous computation and memory footprint of the Transformer module make it difficult to meet the real-time requirements of vehicular edge computing platforms.

Another common strategy is to simply introduce a high-resolution $P_{2}$ layer into the FPN [22], a technique that has been widely validated to improve spatial localization in recent sensor-based vision applications [23]. In fact, the paradigm of extracting multi-scale fused features is universally adopted across domains to handle extreme scale variations and complex target boundaries. For example, in arbitrary-shaped text detection, models like CM-Net [24], Reinforcement Shrink-Mask [25], and Zoom Text Detector [26] successfully leverage geometric boundary modeling and scale-aware feature adaptation to separate closely packed, morphologically complex instances. However, in vehicular infrared scenarios, introducing a high-resolution $P_{2}$ layer is strictly aimed at capturing blurry thermal blobs of distant vehicles, which inherently brings massive high-frequency background thermal noise (i.e., “noise amplification”). Furthermore, vehicular models must prioritize robust semantic filtering against complex urban clutter and maintain strict edge computing efficiency. Although the Full-Scale architecture proposed in this paper also retains the $P_{2}$ layer to ensure the recall of tiny objects, we do not blindly stack computational power. Instead, through subsequent architectural optimization (SAS-Head), we cleverly resolve the computational burden and noise interference brought by high resolution, achieving an optimal balance between detection accuracy and efficiency.

### 2.3. Lightweight Detection Head Design

As the decision-making terminal of an object detector, the design of the Detection Head is crucial to model performance. Current detection head designs are mainly divided into two categories: Decoupled Heads, represented by YOLOX [27] and YOLOv11 [28]; and Shared Heads, represented by FCOS [29] and RetinaNet.

Decoupled heads achieve extremely high detection accuracy by maintaining independent parameter spaces for each scale ($P_{3},P_{4},P_{5}$), but their parameter count grows linearly with the increase in feature levels. When the high-resolution $P_{2}$ layer is introduced, the parameter redundancy problem of decoupled heads becomes particularly prominent, seriously hindering inference speed. Conversely, shared heads reuse the same set of convolutional weights across all scales, greatly reducing the parameter count, but they often struggle to adapt to the drastic scale variations from tiny objects ($P_{2}$) to large objects ($P_{5}$), leading to optimization difficulties.

Different from the decoupled head designs that focus only on accuracy but suffer from parameter redundancy [27], and traditional shared heads that struggle to effectively suppress background noise in the $P_{2}$ layer [29], the SAS-Head proposed in this paper aims to explore a “third path” where lightweight design and strong semantics coexist. Instead of improving speed through simple parameter pruning, we construct an implicit regularization mechanism by leveraging cross-scale parameter sharing. Unlike FCOS, which struggles to adapt to large scale spans, this mechanism forces deep semantic features ($P_{5}$) to participate in the weight updates of shallow layers ($P_{2}$), thereby utilizing contextual information to verify local features. This design not only fills the gap of existing lightweight detection heads in suppressing false alarms for tiny objects, but also establishes a “Mutual Redemption” balance between the detail preservation of the $P_{2}$ layer and the computational efficiency of the detection head, overcoming the computational bottlenecks faced by traditional methods when extending high-resolution layers.

## 3. Materials and Methods

### 3.1. Overall Architecture

**Figure 1:**
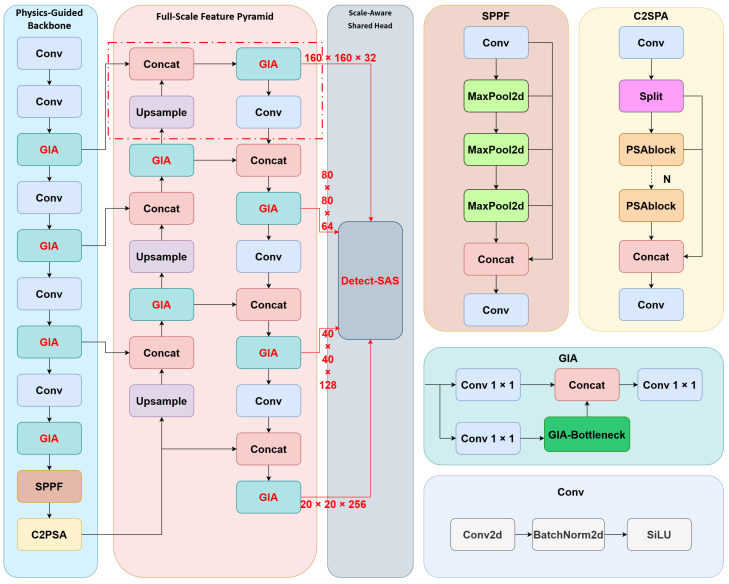
Overview of the Fs2PA. Based on YOLOv11n, this architecture integrates three key components: (1) a physics-guided backbone, which incorporates the GIA module to inject geometric priors; (2) a Full-Scale feature pyramid, which includes a high-resolution $P_{2}$ branch to preserve tiny thermal sources; and (3) an SAS-Head, which achieves synergistic perception from $P_{2}$ to $P_{5}$ through parameter sharing.

To address the two core challenges in vehicular uncooled infrared imaging—namely, “difficulty in feature extraction caused by the lack of texture” and “large-span scale variations (from tiny thermal sources to near-field vehicles)”—this study proposes an Fs2PA, as shown in Figure 1.

The architecture adopts YOLOv11n as the baseline model, constructing an end-to-end closed-loop system that emphasizes both physical perception and semantic enhancement by reconstructing the feature extraction paradigm and detection head topology. Figure 1 intuitively presents the synergistic evolution path of full-scale features across various network levels, which is specifically driven by the following three cascaded key components.

1. Physics-Guided Backbone:
  To overcome the lack of color and texture information in infrared images, we do not directly adopt the native C3k2 modules of YOLOv11. Instead, we embed GIA modules within the backbone network. This module utilizes explicit physical gradient operators to initialize convolution kernels, forcing the network to shift its attention from flat thermal radiation regions to the geometric boundaries of the objects. This design injects a “geometric prior” at the early stages of feature extraction, effectively countering the thermal blurring and low contrast inherent to infrared imagery, and providing the subsequent feature pyramid with structurally rich shallow-layer features.
2. Full-Scale Feature Pyramid:
  Given the significant “micro-scale shift” phenomenon in vehicular scenarios, standard three-scale ($P_{3}$–$P_{5}$) neck networks lead to severe missed detections of long-distance tiny objects. To this end, we extend the topological depth of the PANet to construct a Full-Scale feature pyramid that includes a $P_{2}$ detection layer (Stride = 4). The introduction of this high-resolution branch allows the network to preserve the complete geometric contours of tiny thermal sources (<16×16 pixels), avoiding the “feature erosion” caused by deep downsampling. Meanwhile, we retain the deep $P_{5}$ branch to maintain comprehensive perception of close-range large-scale objects, ensuring all-domain safety in driving scenarios.
3. Scale-Aware Shared Head:
  To address the computational redundancy introduced by the $P_{2}$ layer and the vulnerability of shallow features to background thermal noise, we designed the SAS-Head. This module abandons traditional independent parameter designs and employs a unified set of shared convolutional weights to simultaneously process all feature levels from $P_{2}$ to $P_{5}$. Physically, this mechanism achieves extreme parameter compression, offsetting the computational overhead of the high-resolution layer. Logically, it constructs a cross-scale “semantic resonance”, forcing the global semantic information from the deep $P_{5}$ layer to participate in the gradient updates of the shallow $P_{2}$ layer. This effectively suppresses high-frequency background false alarms, achieving a dual breakthrough in computational efficiency and detection robustness.

In summary, this architecture is not a mere stacking of modules but a closed-loop system. The GIA module effectively tackles the problem of feature blurring in thermal crossover scenarios by injecting geometric priors. The $P_{2}$-Neck successfully retrieves the geometric presence of tiny objects. The SAS-Head, while achieving synergistic perception across all scales, cleverly resolves the conflict between the computational crisis introduced by high resolution and the false alarms caused by shallow noise.

### 3.2. The Gradient-Informed Attention (GIA) Module

Although the standard modules in YOLOv11 (e.g., C3k2 and C2f) extract features efficiently, their data-driven random initialization imposes a strong “texture bias” that prioritizes high-frequency details over global geometric shapes [12]. This inductive bias becomes a severe structural deficiency in TIR imaging, which inherently lacks color and texture [30]. Critically, during “thermal crossover” events—where targets and backgrounds reach thermal equilibrium—conventional texture-reliant convolutions are highly susceptible to overfitting background thermal noise, causing the features of faint targets to be completely submerged [5].

**Figure 2:**
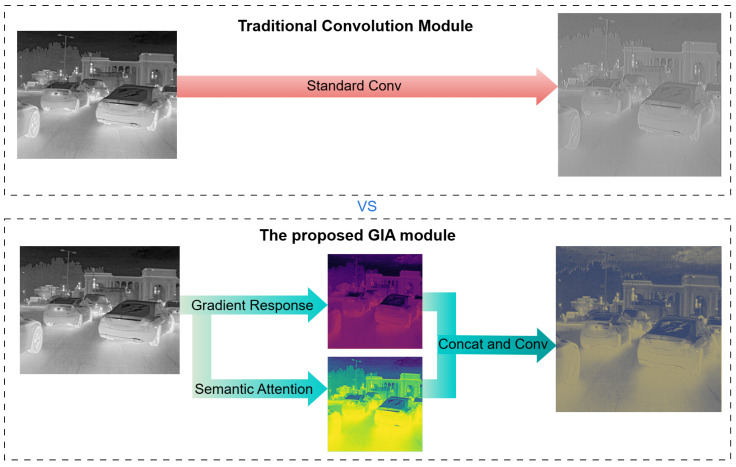
Feature Response Motivation: In thermal crossover scenarios, traditional convolutions (**Top**) are susceptible to background noise interference, whereas GIA (**Bottom**) successfully focuses on the geometric boundaries of the objects by leveraging physical guidance.

To address these challenges, we propose the GIA module to reintroduce explicit geometric priors into the deep network. As illustrated in Figure 2, while traditional convolutions produce blurred feature maps in thermal crossover scenarios, GIA leverages physical gradient operators to prioritize boundary regions, successfully extracting sharp object contours from low-contrast backgrounds. By reinforcing edge gradients, this design fundamentally mitigates a CNN’s texture dependence and significantly enhances its perception robustness toward tiny objects [12,13].

#### 3.2.1. Overall Architecture of GIA

**Figure 3:**
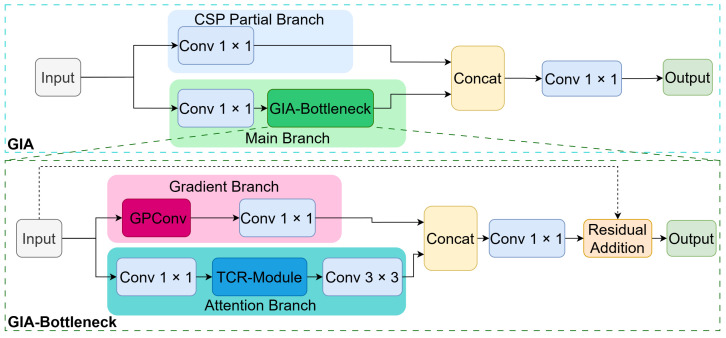
Module Topology: Embedded within the CSP architecture, GIA adopts a dual-stream design of “Gradient-Semantic Parallelism”, comprising the Gradient Branch in the upper path and the Attention Branch in the lower path.

To simultaneously capture explicit physical structures and implicit semantic context, we integrate the GIA module into the Cross Stage Partial (CSP) architecture, replacing standard C3k2 modules. As shown in Figure 3, its core component—the GIA-Bottleneck—employs a dual-branch topology characterized by “Gradient-Semantic Parallelism”.

- Gradient Branch: Dominated by Gradient Prior Convolution (GPConv), this branch acts as a “physical edge detector”. It bypasses pure data-driven learning to extract isotropic gradient magnitudes via Sobel-initialized convolutions, followed by a 1×1 convolution for channel integration.
- Attention Branch: This branch first compresses channels via a 1×1 convolution, then integrates a Target Channel Recalibration (TCR) module [31]. TCR aggregates global spatial context and dynamically recalibrates channel weights via a Multi-Layer Perceptron (MLP), followed by a 3×3 convolution to enhance target-specific features while suppressing thermal noise.

Finally, the dual-branch outputs are concatenated and fused via a 1×1 convolution before being injected into the backbone through a residual connection. This design preserves deep gradient flows while injecting essential geometric priors into the infrared feature stream.

#### 3.2.2. Core Component I: Gradient Prior Convolution (GPConv)

To avoid the slow convergence and noise overfitting associated with random weight initialization in texture-deficient infrared images, GPConv employs isotropic Sobel operators for physics-aware initialization. Instead of calculating gradients independently, it computes the gradient magnitude from standard horizontal ($S_{x}$) and vertical ($S_{y}$) Sobel operators to formulate the initial convolution kernel $K_{init}$:

(1) $$K_{init}=\sqrt{S_{x}^{2}+S_{y}^{2}}\approx \left[\begin{array}{ccc} \sqrt{2} & 2 & \sqrt{2}\\ 2 & 0 & 2\\ \sqrt{2} & 2 & \sqrt{2} \end{array}\right]$$

Unlike directly using frozen kernels, GPConv implements a normalized initialization strategy during the network setup phase to ensure the stable injection of physical priors. The convolutional kernel *W* is strictly normalized as:

(2) $$W_{init}=\frac{K_{init}}{\sum K_{init}}$$

This normalized isotropic initialization serves a dual physical purpose. First, it generates consistent activation responses regardless of edge orientation, allowing the network to capture the complete geometric contours of targets from an ego-centric vehicular perspective. Second, the normalization naturally imparts low-pass filtering characteristics, smoothing flat background regions to effectively suppress the inherent shot noise of uncooled infrared sensors. Finally, the output undergoes Batch Normalization (BN) and SiLU activation for non-linear embedding into the deep network.

#### 3.2.3. Core Component II: Target Channel Recalibration (TCR Module)

While GPConv effectively extracts explicit geometric gradients, these raw signals often contain high-frequency background thermal noise [5,30]. To prevent these contaminated gradients from increasing the false positive rate, the GIA module integrates a TCR mechanism to adaptively filter the channels.

**Figure 4:**
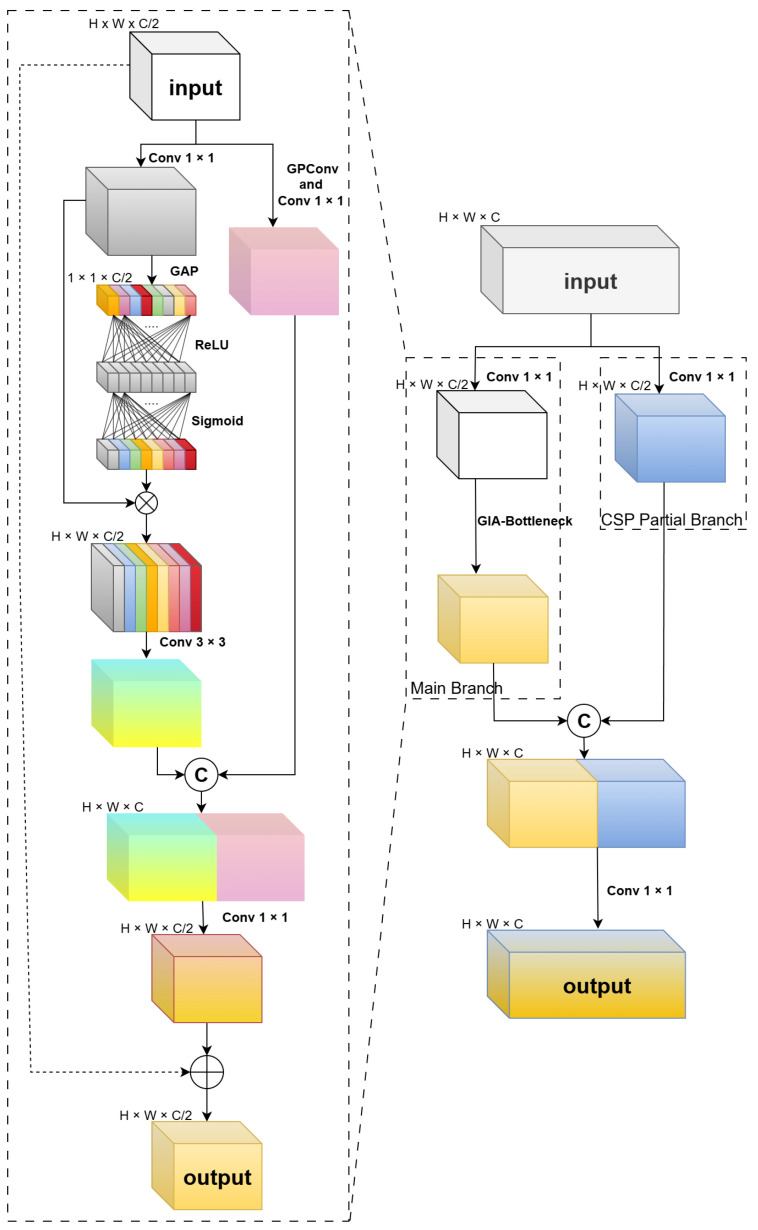
Dimensional Evolution of the GIA Module: This detailedly illustrates the transformation process of the feature tensor within the Attention Branch. The 1×1 channel descriptors pass through the MLP (Conv-ReLU-Conv-Sigmoid) to generate weight vectors, which ultimately achieve adaptive recalibration of the feature maps through a broadcasting mechanism (Scale).

Inspired by Squeeze-and-Excitation networks [31], TCR efficiently models channel dependencies. As illustrated in Figure 4, it first employs Global Average Pooling (GAP) to aggregate global spatial context into a channel descriptor. Subsequently, an MLP consisting of two 1×1 convolutional layers captures nonlinear channel interactions. Notably, we set the channel reduction ratio to r=1 in the MLP to maximize information retention. The MLP outputs a normalized weight vector via a Sigmoid function, which is then applied to the input features through channel-wise multiplication.

This recalibration mechanism adaptively assigns higher weights to channels containing prominent heat sources (e.g., contours of pedestrians and vehicles) while significantly suppressing redundant channels dominated by background clutter. By parallelly fusing explicit geometric gradients with implicit semantic attention, the GIA module ensures that the resulting feature flow possesses both geometric precision and semantic robustness.

### 3.3. Fine-Grained $P_{2}$ Detection Branch

#### 3.3.1. Data Observations and Physical Challenges: Feature Erosion

**Figure 5:**
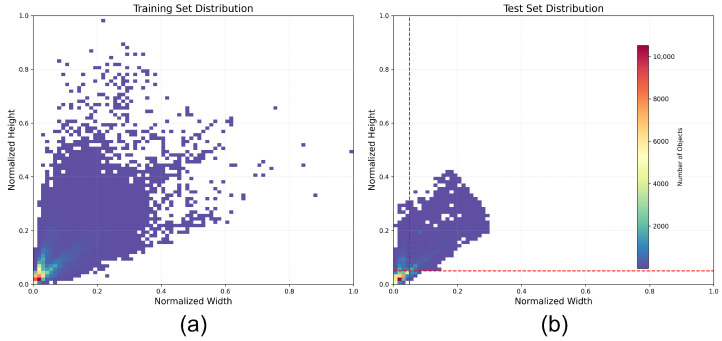
Normalized distribution heatmaps of target scales in the FLIR v2 dataset. (**a**) Normalized target scale distribution heatmap of the training set. (**b**) Normalized target scale distribution heatmap of the test set. The training set exhibits a long-tail distribution, containing some large near-field targets. The test set demonstrates a more extreme “micro-scale shift”, with the vast majority of targets compressed within a minimal range of 0.05 (indicated by the red dashed line). This phenomenon directly drives the design of the Full-Scale pyramid.

Statistical analysis of the FLIR v2 dataset (Figure 5) reveals an extreme “micro-scale shift”, with most targets compressed below a normalized scale of 0.05. However, the vehicular ego-centric view simultaneously captures massive near-field objects. This immense scale span necessitates maintaining the complete $P_{2}$ to $P_{5}$ feature spectrum to ensure exquisite local perception for tiny thermal sources without sacrificing the deep receptive fields required for near-field driving safety.

**Figure 6:**
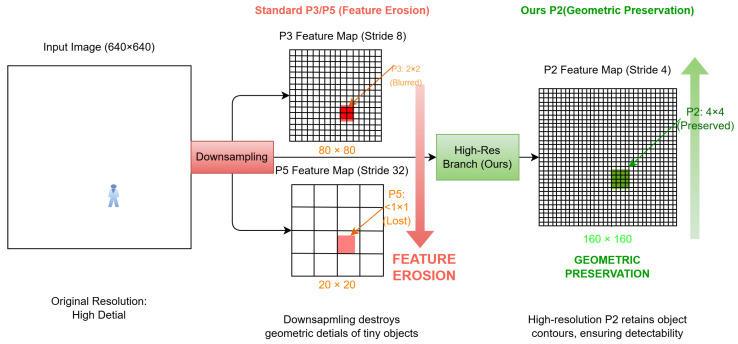
Feature erosion phenomenon caused by convolutional downsampling. Pixel grid analysis reveals that a 16×16 tiny heat source is compressed to the point of geometric information annihilation (sub-pixel level) in the $P_{3}$ (Stride = 8) and $P_{5}$ (Stride = 32) layers. After introducing the $P_{2}$ (Stride = 4) layer, the target is preserved as a clear 4×4 region, maintaining an identifiable geometric contour.

Due to the “Feature Erosion” phenomenon caused by convolutional downsampling, the standard $P_{3}$ layer cannot satisfy the detection requirements for tiny objects. As illustrated by the pixel grid analysis in Figure 6, a $P_{3}$ layer (Stride = 8) compresses a typical 16×16 pixel heat source into a blurred 2×2 block, losing its geometric contour, while the deeper $P_{5}$ layer (Stride = 32) annihilates it entirely to the sub-pixel level. In contrast, the introduced $P_{2}$ layer (Stride = 4) halves the downsampling rate, successfully preserving the target as a clear 4×4 region. This geometric preservation enables the network to effectively distinguish tiny targets from the background using edge gradients, physically confirming the necessity of a high-resolution, Full-Scale branch.

#### 3.3.2. Architectural Implementation

To reverse the aforementioned spatial information loss, we constructed a dedicated high-resolution detection branch—the $P_{2}$ detection layer. Specifically, we extract a new feature path from Stage 2 of the backbone. This layer has a downsampling stride of only 4 (Stride = 4), with a resolution of 1/4 of the input image. Compared to the standard $P_{3}$ layer, the $P_{2}$ layer possesses 4× the pixel density, which effectively maintains the identifiable geometric contours of tiny targets.

To enhance the semantic representation capability of these high-resolution features, we extended the PANet in the Neck section. We perform 2× upsampling on the semantic features of the $P_{3}$ layer and then apply a lateral connection with the shallow features of the backbone’s C2 layer. This design aims to achieve a synergistic feature representation.

- Geometric Details: Directly inherits the rich edge and texture information from the C2 layer (benefiting from the gradient enhancement of the GIA module).
- Initial Semantics: Integrates contextual information from deeper layers.

However, while the simple introduction of the $P_{2}$ layer recovers the geometric presence of tiny targets, it also introduces a new conflict: the high resolution of the $P_{2}$ layer brings massive computational overhead, and the lack of deep semantic constraints makes it highly susceptible to high-frequency background false positives. This prompted us to further design the SAS-Head to achieve the unification of computational efficiency and semantic constraints.

### 3.4. The Scale-Aware Shared Head (SAS-Head)

#### 3.4.1. Design Motivation and Core Mechanism

Although the high-resolution $P_{2}$ layer effectively preserves tiny targets, it introduces a severe “dual-dilemma”: an exponential surge in computational overhead (FLOPs) that challenges edge deployment, and an increased vulnerability to background thermal noise due to insufficient deep semantic constraints.

To break this “zero-sum game” between computational power and precision, inspired by the efficient design of FCOS [29] and the recent lightweight shared architectures (e.g., LSCD [32]), we design the SAS-Head tailored for infrared thermal characteristics. Unlike traditional decoupled heads that maintain independent parameter spaces ($W=\{W_{2},W_{3},W_{4},W_{5}\}$) for each feature level, SAS-Head employs a Cross-Scale Parameter Sharing mechanism using a unified set of convolutional kernels ($W_{share}$). This mathematically constructs a potent multi-scale regularization dynamic; whereas decoupled heads update parameters $W_{i}$ solely based on the local loss $L_{i}$—making the model susceptible to scale-specific overfitting:

(3) $$W_{i}^{\left(t+1\right)}\leftarrow W_{i}^{\left(t\right)}-\eta \cdot \frac{\partial L_{i}}{\partial W_{i}}$$

In SAS-Head, the unique shared weight $W_{share}$ must simultaneously satisfy the optimization objectives of all scales during backpropagation. Its gradient update follows the aggregation of the full-scale gradient flow:

(4) $$W_{share}^{\left(t+1\right)}\leftarrow W_{share}^{\left(t\right)}-\eta \cdot \sum_{i=2}^{5}\lambda_{i}\frac{\partial L(P_{i})}{\partial W_{share}}$$

where $\lambda_{i}$ represents the balancing coefficients for each scale. In our empirical implementation, to avoid redundant hyperparameter tuning and fully leverage the baseline’s data-driven dynamic label assignment mechanism, $\lambda_{i}$ is uniformly set to 1.0. Consequently, the gradient contribution from each level is not rigidly constrained by artificial weights, but rather adaptively balanced by the actual scale distribution of the matched positive samples during training. Equation (4) demonstrates that the update direction of $W_{share}$ is the vector sum determined by the gradient flow propagating from the global semantics of $P_{5}$ down to the fine-grained spatial details of $P_{2}$. This implies that the gradients from the $P_{5}$ layer effectively act as a regularization term for the optimization of the $P_{2}$ layer, forcing the convolutional kernels to learn a scale-invariant representation. This “Mutual Redemption” between feature levels allows the model to achieve anti-noise robustness surpassing that of independent detection heads while significantly reducing parameter redundancy.

#### 3.4.2. Module Architecture and Implementation

**Figure 7:**
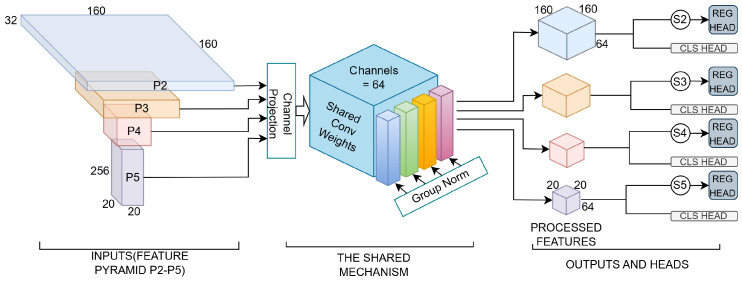
3D Tensor Flow of the SAS-Head: This illustrates how full-scale features ($P_{2}$ to $P_{5}$) are processed through a unified Shared Depthwise Separable Convolution. The blue Shared Weights modules serve as the core hub, which, in coordination with Group Normalization (GN) and Learnable Scale Layers ($\times S_{i}$), achieve cross-scale semantic resonance and extreme parameter compression.

SAS-Head is not a simple mathematical reuse of weights, but a design deeply rooted in the physical perspective of vehicular vision. Due to the perspective effect in ego-centric scenarios, the same class of targets (e.g., pedestrians or vehicles) appears at vastly different scales based on distance—captured by $P_{2}$ when distant and $P_{5}$ when near. Despite the resolution discrepancy, their intrinsic structural semantics and thermal radiation priors remain identical. SAS-Head forces a unified Shared Convolution to extract these scale-invariant essential features. As illustrated in Figure 7, to mathematically resolve the numerical distribution gap across different levels and enable the shared kernel to process them robustly, SAS-Head integrates the three key technologies detailed below.

A. Shared Depthwise Separable Convolution:

To mitigate the computational crisis introduced by the $P_{2}$ layer, we reconstructed the shared processing module into a depthwise separable convolution (Depthwise + Pointwise). This design decouples the convolution into two steps—spatial filtering and channel fusion—reducing computational complexity to approximately 1/9 of standard convolutions. More importantly, as a common processor for full-scale features, this module is compelled to seek a scale-invariant representation that is insensitive to scale variations, serving as the physical carrier for the aforementioned “semantic resonance” and “Mutual Redemption”.

B. Group Normalization (GN) for Cross-Scale Distribution Adaptation:

The primary obstacle to shared weights is the huge distribution gap between shallow and deep layers. Specifically, shallow $P_{2}$ features are primarily composed of high-frequency textures, while deep $P_{5}$ features are dominated by low-frequency semantic blocks, resulting in significantly different mean and variance distributions. Traditional BN attempts to fit this multimodal distribution with a single set of global statistics, which easily leads to training instability. Consequently, we introduced Group Normalization (GN) [33] to bridge this gap. By normalizing within groups of channels, GN’s statistics are independent of spatial scale and batch size. This “instance-level” normalization strategy eliminates the covariate shift caused by inconsistent feature distributions across levels, ensuring that the same shared kernel can robustly handle multi-scale inputs.

C. Scale-Aware Regression Calibration:

Although shared convolutions unify the feature space, object detection regression tasks involve an inherent conflict in numerical scale ranges; in particular, the $P_{2}$ layer is responsible for tiny targets with typically small regression offsets, while the $P_{5}$ layer handles large targets with values an order of magnitude higher. Forcing the same set of weights to fit such vastly different numerical ranges causes convergence difficulties. Therefore, we introduced a learnable scalar coefficient $s_{i}$ (initialized to 1.0) at the end of the regression branch. This coefficient acts as an adaptive “scaling factor”, allowing the model to dynamically rescale the output range for each feature level based on shared features, precisely adapting to the distance prediction requirements of different receptive fields [29].

## 4. Results and Discussions

### 4.1. Datasets and Evaluation Metrics

#### 4.1.1. Dataset Description

##### FLIR v2 Dataset (Primary Dataset)

To evaluate the effectiveness of the proposed method in real-world edge-sensing scenarios, this study utilizes the Teledyne FLIR ADAS Thermal Dataset v2 [34] as the experimental benchmark. As a gold-standard public dataset in the field of vehicular infrared perception, FLIR v2 provides high-resolution (640×512) uncooled thermal imaging data, covering a wide range of complex driving environments such as urban streets, highways, and rural roads.

**Figure 8:**
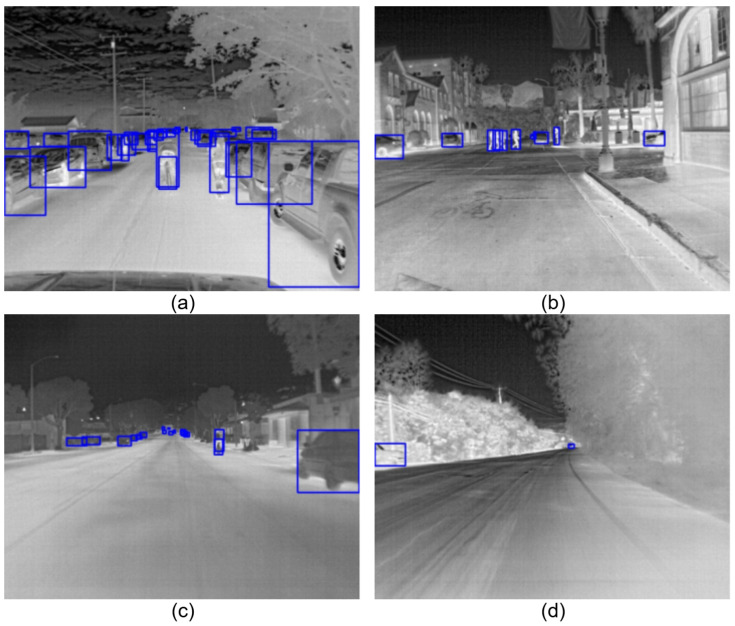
Representative scenarios from the FLIR v2 dataset. The dataset covers diverse environmental conditions, including (**a**) crowded urban streets, (**b**) low-contrast nighttime scenes, (**c**) long-range small objects on highways, and (**d**) complex background clutter with thermal crossover.

Compared with the early v1 version, the v2 version significantly increases scene diversity, with a data distribution of approximately 60% daytime and 40% nighttime scenarios, encompassing various meteorological conditions including clear, cloudy, and hazy weather. These drastic changes in ambient light and temperature lead to severe “thermal crossover” phenomena—where the temperature difference between the target and the background is minimal, resulting in blurred edges. This poses a significant challenge for detectors to extract robust TIR features [2], as illustrated in Figure 8.

To meet the core requirements of autonomous driving obstacle avoidance, we performed label cleaning on the original dataset. To focus on key road safety targets, categories with low relevance to obstacle avoidance tasks were removed. Furthermore, based on the visual similarity in TIR imaging, bicycle and motorcycle were merged into a single unified category. Ultimately, this study selects three critical categories for evaluation: Person, Car, and Bicycle.

Regarding the experimental setup, we followed the official recommended data partitioning standards. The independent video validation sequences provided officially were utilized as the test set to ensure strict spatio-temporal separation between training and testing data. The specific partitioning of the dataset is as follows:

- Training Set: Consists of 10,742 images, used for model parameter optimization.
- Validation Set: Consists of 1144 images, used to monitor model convergence and select the optimal weights during the training process.
- Test Set: Consists of 3749 images sourced from independent video sequences, used to evaluate the model’s generalization capabilities in continuous dynamic scenarios.

In summary, this dataset contains a significant number of occluded objects and long-range tiny targets, providing an objective and comprehensive benchmark to examine the perception performance of lightweight models in complex edge environments.

##### M3FD Dataset (Generalization Dataset)

To further verify the generalization robustness of the proposed model under cross-sensor and cross-scenario conditions, this study introduces the M3FD (Multi-Modal Multi-Scenario Dataset) [6] for zero-shot evaluation. Compared with FLIR v2, the M3FD dataset was collected by different hardware systems and focuses on harsh sensing environments.

**Figure 9:**
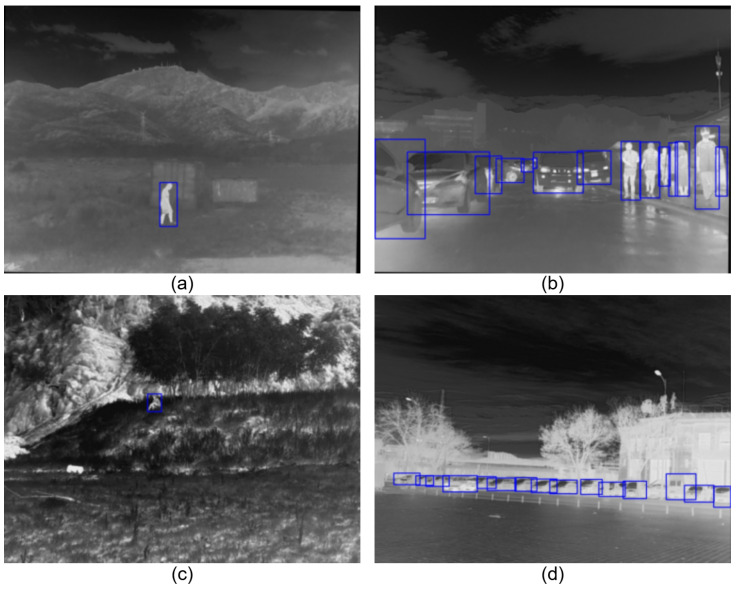
Representative scenarios from the M3FD dataset. The dataset covers extreme environmental degradations including (**a**) heavy smoke obscuration, (**b**) rainy weather conditions, (**c**) unstructured mountainous terrain with concealed targets, and (**d**) complex background interference where vehicle signatures merge with high-radiation buildings.

This dataset contains 4200 registered high-resolution TIR images. Its distinguishing feature is the coverage of various atypical driving scenarios, including heavy fog, thick smoke, and rainy days. Unlike conventional scenarios, these extreme environments introduce complex background thermal disturbances and uncontrolled environmental noise. As shown in Figure 9, in these scenarios, the detector must not only overcome the domain shift caused by differences in sensor specifications but also deal with the potential masking of target features by high-radiation interference sources (such as thick smoke and high-temperature road surfaces). Such complex and variable open-world scenarios can effectively test the robustness of the GIA module’s feature extraction under non-ideal distributions.

To ensure that the test data aligns strictly with the category definitions of the source domain (FLIR v2), we performed label cleaning and mapping on the original M3FD annotations:

- Class Mapping: The original label “People” was mapped to Person, and “Motorcycle” was mapped to Bicycle.
- Class Filtering: Considering that the thermal signatures of Bus and Truck in M3FD differ significantly from the Car category (primarily sedans) defined in FLIR v2, we treated categories such as Bus, Truck, and Lamp as background and removed them to avoid evaluation errors caused by definition ambiguity. Only the original “Car” label was retained for the Car category.

Ultimately, to objectively evaluate the cross-domain generalization performance of our model, we directly tested the model trained on FLIR v2 on this dataset without any fine-tuning.

#### 4.1.2. Evaluation Metrics

To comprehensively evaluate the proposed method, we adopt the standard COCO benchmark evaluation protocol [10]. The system’s performance is assessed across two dimensions: detection accuracy and model complexity.

For detection accuracy, we utilize Precision (*P*), Recall (*R*), and mean Average Precision (mAP) [1,35]. Specifically, mAP@50 denotes the average precision at an Intersection over Union (IoU) threshold of 0.5, reflecting the basic classification and bounding box localization capabilities. To rigorously assess the model’s robustness in high-precision localization, we also report mAP@50:95, which averages the mAP over IoU thresholds ranging from 0.5 to 0.95 (with a step size of 0.05).

To evaluate the feasibility of edge deployment, we measure the model’s computational complexity and inference speed using Giga Floating-point Operations (GFLOPs) and Frames Per Second (FPS). GFLOPs quantifies the theoretical computational overhead of a single forward pass, while FPS directly reflects the end-to-end system throughput, encompassing pre-processing, network inference, and post-processing latency.

### 4.2. Experimental Environment and Implementation Details

To ensure the fairness and reproducibility of the experimental results, all experiments in this study were conducted on a unified high-performance computing platform.

Hardware and Software Setup

The experimental workstation is equipped with a server-grade AMD EPYC 7K62 48-Core Processor (Advanced Micro Devices, Inc., Santa Clara, CA, USA) and a high-performance NVIDIA GeForce RTX 4090 D GPU (NVIDIA Corporation, Santa Clara, CA, USA) with 24 GB of VRAM. The system memory (RAM) is 90 GB, which is sufficient to support parallel loading and processing of large-scale infrared datasets. The operating system is based on the Linux (Debian 6.1) distribution. The software environment is built on the Python 3.10 programming language, with PyTorch 2.2.2 as the deep learning framework. To fully exploit the hardware’s computational power, we configured the CUDA 12.1 platform and the CuDNN 8.9.0 library for deep neural network acceleration, enabling efficient model training and inference.

Training Strategy

All models were trained in an end-to-end manner, with the input image resolution uniformly resized to 640×640 pixels. No pre-trained weights were utilized; all parameters were trained from scratch to ensure models fully adapt to the unique thermal radiation distribution of infrared images without the implicit bias of visible-light transfer learning.

For the proposed architecture and YOLO-series baselines, the Stochastic Gradient Descent (SGD) optimizer was employed with an initial learning rate of $1\times 10^{-2}$ (linear decay to 1%), a momentum of 0.937, and a weight decay of $5\times 10^{-4}$. Crucially, to avoid artificially suppressing the performance of heterogeneous baseline models (e.g., CenterNet [36], D-FINE [37]), we adopted their officially recommended optimizers (such as AdamW) and optimal learning rates to ensure they achieved their maximum potential.

Implementation and Evaluation Details

The training pipeline was standardized with a total batch size of 32 for 300 epochs. An early stopping mechanism was introduced, terminating training if the validation mAP failed to improve for 50 consecutive epochs. To enhance robustness against scale variations and thermal clutter, a composite augmentation strategy (Mosaic, Copy-Paste, Random Erasing) was tailored for the proposed method and YOLO baselines. The strong Mosaic augmentation was disabled for the last 10 epochs to fit the authentic data distribution. Models inherently incompatible with these augmentations followed their standard pipelines. The global random seed was fixed to 42.

To ensure a rigorous and transparent evaluation of model efficiency, the reported FPS metric measures the strict end-to-end throughput, encompassing pre-processing, model forward propagation, and post-processing (NMS). While the primary comparative experiments report FPS under a standardized setting (Batch Size 16, FP16 half-precision), we conducted a comprehensive latency benchmarking under varying precision formats (FP32 vs. FP16) and batch sizes (BS = 1 vs. BS = 16) to ensure full reproducibility, as detailed in Table 1. All inference evaluations were strictly executed on a single NVIDIA RTX 4090 D GPU.

### 4.3. Ablation Studies

To comprehensively verify the effectiveness of the proposed Fs2PA, we conducted a series of incremental ablation experiments on the FLIR v2 dataset. These experiments focus not only on the absolute improvement in detection accuracy (mAP, but also emphasize evaluating the performance of each component in terms of the accuracy–efficiency trade-off. We established YOLOv11n as the baseline model to sequentially examine the contributions of the GIA module, the Full-Scale $P_{2}$ Detection Layer, and the SAS-Head.

#### 4.3.1. Progressive Effectiveness Analysis

**Figure 10:**
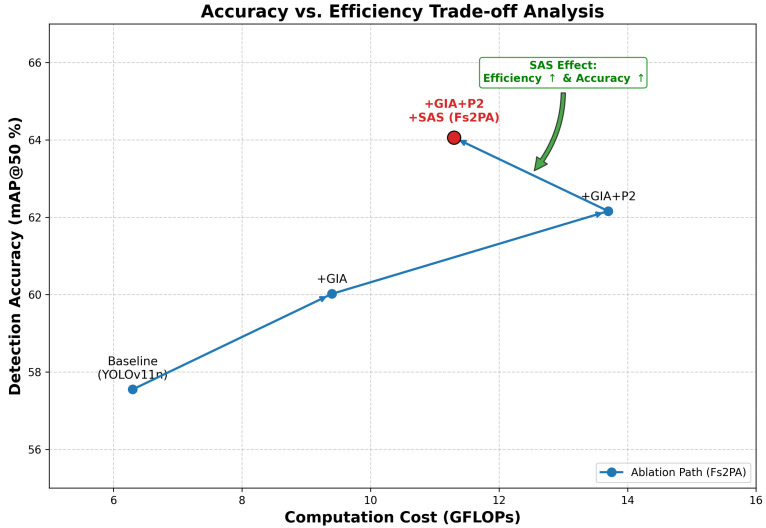
Ablation path analysis visualizing the trade-off between accuracy and computational cost. The proposed method (red star) achieves a Pareto-optimal balance, significantly reducing GFLOPs compared to the $P_{2}$-only baseline while maximizing mAP.

Table 2 provides a detailed listing of the quantitative metrics at each stage, while Figure 10 intuitively illustrates the evolution trajectory of the model within the “Accuracy (mAP) vs. Computational Complexity (GFLOPs)” coordinate system.

(1) GIA Module:

As shown in the second row of Table 2, integrating only the GIA module into the backbone improves the mAP@50 from 57.55% to 60.02% (+2.47%). This significant gain validates the critical role of “geometric priors” in infrared imaging. Since infrared images often suffer from blurred boundaries due to thermal crossover [5], GIA breaks the standard CNN’s texture bias through normalized gradient initialization, enabling the model to acutely capture faint edge contours even in low-contrast scenarios. Notably, this improvement introduces only a minimal computational overhead (increasing from 6.3 to 9.4 GFLOPs). This demonstrates that GIA possesses an extremely high “return on investment”, serving as the most efficient means of addressing the problem of blurred discriminative features.

(2) Full-Scale $P_{2}$ Layer:

To resolve the “micro-scale shift” problem encountered in vehicular perspectives, we introduced the high-resolution $P_{2}$ layer. This enhancement further pushed the mAP@50 to 62.16% (+2.14%), proving that recovering spatial details lost during downsampling is vital for detecting long-range, minute thermal sources—a finding consistent with the observations in TPH-YOLOv5 [21]. However, as illustrated in Figure 10, this step caused the computational complexity to surge to 13.7 GFLOPs (+45%). This validates the “computational overhead” challenge mentioned in the Introduction: while simply stacking high-resolution layers restores the geometric presence of tiny targets, it severely sacrifices inference efficiency, pushing the model away from the optimal operating zone for edge device deployment.

(3) SAS-Head:

Following the replacement of standard decoupled heads with SAS-Head, a significant “pullback effect” occurred, as indicated by the red arrow in Figure 10. The inclusion of SAS-Head dramatically reduced the computational load from 13.7 back to 11.3 GFLOPs (−17.5%), effectively neutralizing the computational crisis introduced by the $P_{2}$ layer and aligning the model with the real-time requirements of edge computing. Despite the substantial reduction in parameters, the mAP@50 counter-intuitively climbed further to 64.06%. This demonstrates that the SAS module is not merely a model compression tool, but a “collaborative enhancer”. Through cross-scale parameter sharing, it forces the network to learn a compact and robust feature representation, eliminating parameter redundancy in the decoupled heads while suppressing background noise in the $P_{2}$ layer via deep semantic constraints. Ultimately, our complete architecture achieves an optimal Pareto Frontier between accuracy and efficiency.

#### 4.3.2. Architectural Paradigm Analysis: The Paradox of $P_{5}$

To delve deeper into the internal mechanisms of the SAS-Head—specifically, how it resolves the issue of multi-scale feature conflict—we designed a set of comparative experiments regarding the utilization of deep-layer features ($P_{5}$) (see Table 3).

(1) The Dilemma of Standard Heads: $P_{5}$ as a “Noise Source”

When using a Standard Decoupled Head, we observed an anomalous phenomenon: removing the $P_{5}$ layer (Model B) actually achieved a slightly higher mAP@50 compared to the Full-Scale model that retains $P_{5}$ (Model A) (62.24% vs. 62.16%).

This indicates that under the standard convolutional paradigm lacking strong constraints, the low-resolution $P_{5}$ layer (Stride = 32) fails to provide effective semantic information for infrared small object detection. Instead, it introduces excessive background thermal radiation noise. In this context, the $P_{5}$ layer effectively becomes a burden to the system, which explains why some lightweight infrared detection networks tend to discard deep-layer features or employ complex attention mechanisms to alleviate this issue.

(2) Semantic Activation of SAS: $P_{5}$ as a “Semantic Anchor”

However, the introduction of SAS-Head fundamentally changes this situation, successfully transforming $P_{5}$ from a redundant component into a beneficial constraint.

- Without $P_{5}$ (Model C): When applying SAS to the $P_{2}$–$P_{4}$ architecture, performance drops significantly to 61.44%. This suggests that without the global context provided by the deep-layer hierarchy, it is difficult for shared weights to converge to a state that is robust across all scales.
- With $P_{5}$ (Model D-Fs2PA): When $P_{5}$ is retained, SAS utilizes its global semantic information to guide the update of shared features, pushing the mAP up to 64.06%.

This comparison strongly demonstrates that the SAS module establishes a “Semantic Resonance” mechanism. It forces the use of the global contextual information from $P_{5}$ to “calibrate” and “filter” the local features of the shallow $P_{2}$ layer, thereby resolving the paradox where $P_{5}$ is both necessary for semantic guidance and detrimental due to noise. Through this design, the SAS-Head successfully activates the latent potential of deep-layer features, achieving effective collaboration across full-scale features.

### 4.4. Comparative Experiments

To verify the comprehensive performance of the proposed algorithm in infrared vehicular scenarios, we conducted a Full-Scale comparison between Fs2PA and current mainstream real-time object detectors on the FLIR v2 test set. To provide a domain-relevant and exhaustive assessment, the comparative targets encompass two major categories: (1) General-purpose advanced detection paradigms, including the classic anchor-free CenterNet [36], extreme decoupled-head YOLOX [27], cutting-edge Transformer-based detectors like RT-DETR [38] and D-FINE [37], and the full evolutionary lineage of the YOLO series (ranging from YOLOv5 [39] to YOLOv13 [40]). (2) Established infrared-specific detection methods, including attention-enhanced models tailored for thermal imaging such as YOLOv8-CBAM [41,42], YOLOv8-CA [43,44], YOLOv8-IRD [45], and YOLO-FIRI [46]. Notably, YOLOv11 [28] serves as the primary baseline for this experiment. The experimental design is bifurcated into two parts: intra-scale competitive benchmarking (Nano-scale) and cross-scale performance dominance. Furthermore, zero-shot generalization tests were conducted on the M3FD dataset [6] to evaluate the model’s advantages in parameter efficiency and generalization capability from all dimensions.

Disclaimer regarding preprints: It is noted that certain cutting-edge models included in our benchmarking (e.g., YOLOv13 and D-FINE) are currently available only as arXiv preprints. While they are included to provide the most up-to-date technological trajectory, their architectural claims have not yet undergone formal peer review, and comparative conclusions against them are drawn with corresponding academic caution.

#### 4.4.1. Intra-Scale Comparison (Nano-Scale)

Table 4 presents the performance comparison between our method and other nano-scale models on the FLIR v2 dataset. All of these models are designed with the goal of extremely low computational resource consumption, making them suitable for deployment on edge devices.

As shown in Table 4, standard lightweight detectors (e.g., YOLOv10n) and their latest iterations encounter a critical efficiency–accuracy trade-off, plateauing around 57% mAP@50 due to severe feature erosion. Conversely, despite mitigating the quadratic complexity of self-attention, Transformer-based architectures (RT-DETR-N, D-FINE-N) still suffer from significant memory-bound latency overhead, rendering them unviable for strict real-time deployment. Furthermore, established infrared-specific methods (e.g., YOLOv8-CBAM, YOLO-FIRI) relying on simple attention injections fail to structurally resolve the “micro-scale shift”, also stalling below the 57.5% mAP@50 ceiling. In stark contrast, Fs2PA fundamentally breaks this performance bottleneck. By integrating the explicit geometric priors of GIA and the semantic resonance of SAS-Head, our model pushes the state-of-the-art mAP@50 to 64.06%—outperforming the baseline YOLOv11n by 6.51%. More importantly, our synergistic design neutralizes the computational overhead of the high-resolution $P_{2}$ layer, maintaining a highly efficient inference speed of 547 FPS, thereby achieving the optimal Pareto frontier for real-world vehicular perception.

#### 4.4.2. Cross-Scale Comparison

To further verify the efficiency ratio of the proposed architecture, we conducted a “cross-scale challenge” by comparing our Nano-scale model (11.3 GFLOPs) against the small-scale models (typically > 20 GFLOPs) of the previous generation.

The experimental results (Table 5) demonstrate that our Nano-scale model comprehensively outperforms the Small-scale competitors in terms of accuracy. Specifically, Fs2PA (64.06%) is significantly superior to YOLOv8s (62.27%) and YOLOv11s (61.59%). This superiority is achieved even with the computational load being halved; for instance, the computational cost of YOLOv8s is 28.4 GFLOPs, which is 2.5 times that of our model. This counter-intuitive result provides strong evidence that in infrared small object detection tasks, the gains brought by simply increasing the number of channels (Channel Scaling—the conventional upgrade path from Nano to Small) are far less efficient than those gained by optimizing feature scales (introducing $P_{2}$) and improving feature fusion mechanisms (introducing SAS). By addressing the core pain point of “micro-scale shift”, our architecture unlocks superior performance compared to the blind stacking of parameters.

### 4.5. Generalization Analysis

To verify the robustness of the model across different sensors and environmental conditions, we conducted Zero-shot Evaluation on the M3FD (Multi-Modal Multi-Scene Multi-Label) dataset [6], which was not involved in the training process.

As shown in Table 6, standard YOLO models generally experienced performance degradation during cross-domain testing, with the baseline YOLOv11n dropping to 53.96% mAP@50. Crucially, even infrared-specific models equipped with attention mechanisms (e.g., YOLOv8-CBAM at 54.20% and YOLOv8-IRD at 53.40%) failed to demonstrate robust cross-domain adaptability, indicating an overfitting tendency toward the source domain’s specific thermal style. In contrast, Fs2PA maintained a high accuracy of 57.94%, leading the baseline by nearly 4%. This superior generalization capability is attributed to the synergistic design of the Fs2PA. Specifically, the GIA module focuses on essential geometric contours rather than absolute thermal radiation values, making it inherently insensitive to sensor-induced imaging differences. Simultaneously, the SAS-Head provides robust deep semantic constraints, preventing the network from overfitting to the source domain’s specific thermal distribution.

Furthermore, it is worth noting that the zero-shot evaluation metrics on the M3FD dataset represent a conservative lower bound of the model’s actual performance. During the dataset preprocessing phase, certain categories (e.g., buses and trucks) were treated as background to strictly align with the core driving categories of the source domain. However, since unannotated physical entities still remain in the test images and share highly similar global thermal signatures with the targets, robust detectors may occasionally localize them. Due to the absence of corresponding ground truth annotations, these logically reasonable semantic detections are inevitably penalized as False Positives by the standard evaluation script. This inherent label-filtering mechanism causes a systemic underestimation of the absolute Precision metric, which conversely underscores the model’s strong cross-domain geometric contour recognition capabilities.

### 4.6. Qualitative Analysis

To intuitively verify the effectiveness of the proposed method and reveal the internal decision-making mechanisms of the network at the feature response level, this section provides a qualitative analysis of the detection results and visualizes the underlying mechanisms using Grad-CAM [51].

#### 4.6.1. Qualitative Detection Results

**Figure 11:**
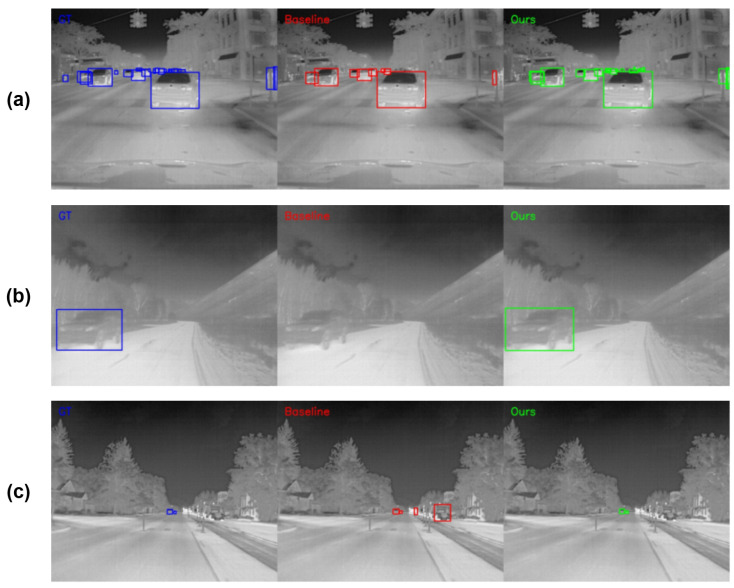
Detection results for three typical scenarios in the FLIR v2 dataset: (**a**) long-range tiny objects; (**b**) boundary dissolution induced by thermal crossover; (**c**) cluttered background and semantic interference. The first, second, and last columns show the Ground Truth, detection results by YOLOv11n, and detection results by the proposed Fs2PA model, respectively.

To objectively measure the robustness of the model in real-world complex scenarios, we selected highly challenging and representative samples from the FLIR v2 test set for visual comparison, as shown in Figure 11.

The comparative analysis covers the three core pain points in infrared vehicular perception:

- Scenario A: Long-range Tiny Objects. As shown in Figure 11a, due to the perspective effect caused by distance, targets occupy very few pixels (<16×16) in the field of view and exhibit blurred textures. As the baseline model’s detection head is based on the $P_{3}$ layer (8× downsampling), the spatial features of tiny objects are severely eroded during deep convolutions, leading to systematic missed detections. In contrast, benefiting from the introduction of the Full-Scale $P_{2}$ Layer, our method successfully preserves high-resolution geometric features from shallow layers. It can acutely capture faint thermal signals and recall the vast majority of tiny objects, significantly outperforming the Baseline’s “blindness” to them.
- Scenario B: Boundary Dissolution Induced by Thermal Crossover. Figure 11b illustrates the severe feature degradation caused by “thermal crossover”. The thermal radiation intensity of the vehicle body highly converges with the background vegetation. This thermal equilibrium, coupled with complex vegetation textures, causes the gradient boundaries to almost disappear, visually “melting” the target into the environment. The Baseline model struggles to separate targets from such low-contrast backgrounds, frequently resulting in missed detections. However, our proposed GIA module enhances the perception of faint contours by injecting explicit gradient priors. Experiments demonstrate that even when target boundaries undergo severe dissolution, our method can still accurately delineate potential targets.
- Scenario C: Cluttered Background & Semantic Interference. Figure 11c presents complex street scenes containing numerous non-target heat sources (e.g., truck structures, road hot spots). Lacking sufficient semantic discriminability, the Baseline model easily misclassifies background noise with similar thermal characteristics as vehicles, leading to a surge in the false alarm rate. Conversely, our model utilizes the Deep Semantic Constraints provided by the SAS-Head to effectively leverage global contextual information for calibrating the logical validity of target categories. It successfully filters out false alarms caused by background hot spots and semantic confusion, demonstrating exceptionally strong anti-interference robustness.

#### 4.6.2. Internal Mechanism Verification via Grad-CAM

**Figure 12:**
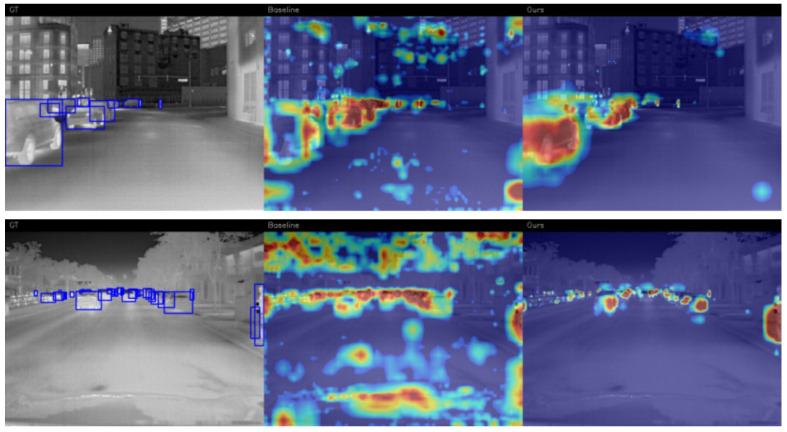
Grad-CAM visualization comparisons under typical challenging scenarios from the FLIR v2 dataset. The first, second, and last columns represent the original thermal images, the attention heatmaps of the Baseline (YOLOv11n), and the attention heatmaps of the Proposed Model, respectively. The red highlighted regions indicate the key features the network focuses on during the decision-making process.

To deeply analyze the reshaping effect of the Fs2PA on the network’s attention mechanism—and specifically to verify the noise suppression capability of the GIA module and the deep semantic activation effect of the SAS module—we utilized Grad-CAM [51] to conduct a visual analysis of the final feature map of the detection head, as shown in Figure 12.

As illustrated in the urban street and open road scenarios, the Baseline model exhibits significant scattered feature activations. Its thermal response suffers from severe semantic drift, falsely activating on background areas with similar thermal characteristics (e.g., building windows and ground reflections) and presenting indistinguishable ambiguous activation regions under large-scale thermal interference from the sky and road surface. In contrast, the proposed model, driven by the GIA module and SAS-Head, leverages explicit edge gradient priors and deep semantic constraints to effectively decouple the main attention from environmental noise. Although trace residual responses exist in the background, the primary highlighted centers are precisely constrained within the geometric contours of the targets. This synergistic mechanism not only completely suppresses the high-intensity false activations caused by background clutter, but also acutely locks onto the geometric boundaries of physical entities even under severe thermal radiation interference. Consequently, it successfully preserves instance-level feature granularity and effectively mines discriminative regions in low-contrast environments, demonstrating exceptional robustness for vehicular perception.

### 4.7. Discussions

Regarding the “lightweight” paradox, while Fs2PA increases the computational cost to 11.3 GFLOPs compared to the raw YOLOv11n baseline (6.3), this is a necessitated design trade-off. Detecting severely degraded thermal targets physically requires the high-resolution $P_{2}$ layer, which natively surged the cost to 13.7 GFLOPs. The SAS-Head effectively neutralized this overhead, compressing the final footprint back to 11.3 GFLOPs. Consequently, although categorized as a Nano-scale (N) model, Fs2PA punches above its weight class. It comprehensively outperforms previous-generation Small-scale (S) architectures (e.g., YOLOv8s, YOLOv11s) in accuracy while utilizing less than half their computational resources (∼28 vs. 11.3 GFLOPs). This proves that optimizing feature scales and fusion mechanisms is fundamentally more efficient than blindly stacking network width.

Practically, Fs2PA is positioned for vehicular edge perception rather than extreme ultra-low-power microcontrollers (MCUs). It delivers a blazing throughput of 547 FPS on a high-end RTX 4090 D GPU. More importantly for its target operational environment, its 11.3 GFLOPs footprint—when paired with standard edge acceleration (e.g., TensorRT FP16)—demonstrates the theoretical feasibility to support real-time inference (∼30 FPS) on mainstream power-constrained vehicular platforms like the NVIDIA Jetson Orin Nano (<15W). Despite this optimal hardware–accuracy balance, the indispensable $P_{2}$ layer sets a hard theoretical lower bound, precluding deployment on highly resource-deprived MCUs. Additionally, while highly robust to thermal crossover, extreme weather conditions (e.g., torrential rain or thick mud) that physically obliterate thermal radiation expose the inherent limitations of single-modality perception. Future work will explore lightweight multi-modal fusion strategies, such as integrating millimeter-wave radar, to maintain edge-friendly efficiency under absolute visual degradation.

## 5. Conclusions

Addressing the two primary challenges of “texture deficiency” and “micro-scale shift” in vehicular infrared imaging, this study proposes the Fs2PA. This architecture innovatively integrates the three following core components:

- The GIA module injects explicit geometric priors through normalized gradient initialization and attention recalibration, thereby breaking the traditional “texture bias” to acutely capture blurred boundaries in thermal crossover scenarios.
- A Full-Scale feature pyrami introduces a high-resolution $P_{2}$ branch to reverse the feature erosion caused by deep downsampling, effectively preserving the geometric contours of long-range tiny objects.
- The SAS-Head utilizes a cross-scale parameter-sharing mechanism to forge a “semantic resonance”, leveraging deep-layer semantics to suppress shallow-layer background noise while effectively neutralizing computational redundancy.

Extensive experiments on the FLIR v2 and M3FD datasets demonstrate that the proposed method achieves superior performance across multiple dimensions. Specifically, on the FLIR v2 dataset, Fs2PA improves the mAP@50 to 64.06% (an absolute gain of +6.51% over the baseline) while maintaining an ultra-high real-time inference speed of 547 FPS. Furthermore, it demonstrates cross-scale dominance by outperforming previous-generation Small-scale architectures while utilizing less than half the computational cost (11.3 vs. 28.4 GFLOPs). In zero-shot cross-domain evaluations on the M3FD dataset, it maintains a leading mAP@50 of 57.94%, proving its exceptional generalization robustness. Fundamentally, this success stems from the proposed “physics–semantic dual-stream mechanism”, achieving an optimal trade-off between efficiency and accuracy.

In summary, this research provides an efficient and robust infrared detection solution tailored for autonomous driving, establishing a generalized design paradigm that bridges physical feature extraction with deep semantic understanding. By successfully neutralizing the traditional efficiency–accuracy trade-off, Fs2PA provides a viable pathway for highly reliable, real-time vehicular edge perception in complex open-world environments. 

## Figures and Tables

**Table 1:** Inference speed (FPS) benchmarking under different precision formats and batch size settings on a single RTX 4090 D GPU.

Model	Batch Size = 1		Batch Size = 16	
	FP32	FP16	FP32	FP16
Baseline (YOLOv11n)	112.0	113.2	713.9	704.6
Fs2PA (Ours)	70.3	70.3	525.5	547.2

**Table 2:** Ablation studies of the proposed Fs2PA. Step 3 constructs the full-scale features ($P_{2}$–$P_{5}$), and Step 4 introduces the Synergistic head (SAS). The per-class AP@50 metrics are provided to illustrate category-specific performance. The checkmark (✓) indicates the integration of the corresponding module. Bold numbers indicate the best performance in each column.

Model	GIA	P2 Layer	SAS-Head	mAP@50 (%)	AP_*Person*_ (%)	AP_*Bicycle*_ (%)	AP_*Car*_ (%)	GFLOPs
Baseline (YOLOv11n)	-	-	-	57.55	72.43	25.35	74.88	6.3
+GIA	✓	-	-	60.02	73.65	29.67	76.74	9.4
+P2	✓	✓	-	62.16	76.58	28.92	80.98	13.7
+SAS (Fs2PA)	✓	✓	✓	**64.06**	**77.16**	**33.18**	**81.83**	11.3

**Table 3:** Analysis of the impact of the $P_{5}$ layer and SAS-Head. Bold text indicates the proposed optimal model and the best performance metrics.

Model ID	Feature Scales	Head Type	mAP@50 (%)	mAP@50:95 (%)	GFLOPs	Description
A	P2–P5	Standard	62.16	36.23	13.7	Redundant P5
B	P2–P4	Standard	62.24	36.13	13.0	Naive Light
C	P2–P4	SAS-Head	61.44	36.20	10.7	Incomplete
**D (Fs2PA)**	**P2–P5**	**SAS-Head**	**64.06**	**37.02**	11.3	**Optimal**

**Table 4:** Performance comparison (Nano-scale) on FLIR v2. N/A: Not Applicable; N: Nano; T: Tiny. Bold text indicates the proposed method and the best performance in each column.

Method	Scale	Prec. (%)	Rec. (%)	mAP@50 (%)	mAP@50:95 (%)	GFLOPs	FPS
CenterNet-R18 [36]	N/A	-	-	17.00	5.90	22.5	232
YOLOX-nano [27]	N	-	-	45.90	23.70	2.6	585
YOLOX-tiny [27]	T	-	-	52.20	29.00	15.2	621
RT-DETR-N [38]	N	61.48	46.65	51.45	26.58	26.9	262
D-FINE-N [37]	N	78.12	63.20	57.50	32.00	7.1	87
YOLOv8-CBAM [41,42]	N	70.60	50.80	57.00	32.40	8.1	769
YOLOv8-CA [43,44]	N	72.50	50.40	57.50	33.10	8.1	833
YOLOv8-IRD [45]	N	69.20	54.10	60.60	34.60	10.8	500
YOLO-FIRI [46]	T	60.90	45.70	44.80	20.60	15.2	270
YOLOv5nu [39]	N	69.53	50.31	56.13	31.73	7.1	604
YOLOv8n [47]	N	69.60	51.01	56.75	32.44	8.1	622
YOLOv9t [48]	T	70.69	52.14	58.36	33.03	7.6	408
YOLOv10n [49]	N	68.95	49.17	57.01	32.63	6.5	739
YOLOv11n [28]	N	68.31	51.33	57.55	32.55	6.3	705
YOLOv12n [50]	N	68.32	51.80	57.51	32.57	5.8	651
YOLOv13n [40]	N	69.85	49.81	56.15	30.98	6.1	492
**Fs2PA**	**N**	73.48	56.56	**64.06**	**37.02**	11.3	547

**Table 5:** Cross-scale performance comparison between the proposed Fs2PA (Nano-scale) and state-of-the-art Small-scale models. S: Small; N: Nano. Bold text indicates the proposed method and the best performance in each column.

Method	Scale	Prec. (%)	Rec. (%)	mAP@50 (%)	mAP@50:95 (%)	GFLOPs	FPS
YOLOv5su	S	72.14	55.29	61.77	36.18	23.8	460
YOLOv8s	S	72.87	55.57	62.27	35.94	28.4	424
YOLOv9s	S	72.11	55.82	61.89	36.24	26.7	394
YOLOv10s	S	72.48	53.19	61.80	36.32	21.4	525
YOLOv11s	S	71.32	55.04	61.59	36.32	21.3	552
**Fs2PA**	**N**	73.48	56.56	**64.06**	**37.02**	**11.3**	547

**Table 6:** Zero-shot generalization on M3FD (Nano-scale benchmark). N/A: Not Applicable; N: Nano; T: Tiny. Bold text indicates the proposed method and the best performance in each column.

Method	Scale	Prec. (%)	Rec. (%)	mAP@50 (%)	mAP@50:95 (%)	FPS
CenterNet-R18	N/A	-	-	15.00	5.40	108
YOLOX-nano	N	-	-	38.80	18.30	610
YOLOX-tiny	T	-	-	45.20	24.10	565
RT-DETR-N	N	63.37	47.48	51.85	27.27	256
D-FINE-N	N	73.99	62.31	51.10	27.40	79
YOLOv8-CBAM	N	69.10	49.40	54.20	30.50	769
YOLOv8-CA	N	74.40	48.30	54.40	30.20	833
YOLOv8-IRD	N	69.60	47.90	53.40	29.00	500
YOLO-FIRI	T	58.90	45.90	43.30	19.00	250
YOLOv5nu	N	74.19	46.51	52.91	28.71	554
YOLOv8n	N	70.30	49.05	53.60	29.53	589
YOLOv9t	N	71.06	49.47	54.61	30.01	426
YOLOv10n	N	70.52	48.36	54.19	29.79	709
YOLOv11n	N	70.67	48.85	53.96	29.89	738
YOLOv12n	N	66.56	48.65	53.62	29.74	620
YOLOv13n	N	71.13	47.14	53.23	29.03	488
**Fs2PA**	N	71.40	52.31	**57.94**	**31.89**	524

## Data Availability

Publicly available datasets were analyzed in this study. The FLIR ADAS Thermal Dataset v2 can be accessed at https://adas-dataset-v2.flirconservator.com (accessed on 25 October 2025). The M3FD dataset is publicly available in the authors’ official repository at https://github.com/JinyuanLiu-CV/TarDAL (accessed on 20 January 2026).
